# Exploring Aerobic Energy Metabolism in Breast Cancer: A Mutational Profile of Glycolysis and Oxidative Phosphorylation

**DOI:** 10.3390/ijms252312585

**Published:** 2024-11-23

**Authors:** Ricardo Cunha de Oliveira, Giovanna C. Cavalcante, Giordano B. Soares-Souza

**Affiliations:** 1 Laboratório de Genética Humana e Médica, Pós-Graduação em Genética e Biologia Molecular, Universidade Federal do Pará, Belém 66075-110, Pará, Brazil; oliveira.ca.ricardo@gmail.com; 2 Departamento de Bioquímica, Instituto de Química, Universidade de São Paulo, São Paulo 05508-900, São Paulo, Brazil; 3 Instituto Tecnológico Vale (ITV-DS), Belém 66055-090, Pará, Brazil

**Keywords:** biomarkers, glycolysis, mitochondria, oncogenes, oxidative phosphorylation

## Abstract

Energy metabolism is a fundamental aspect of the aggressiveness and invasiveness of breast cancer (BC), the neoplasm that most affects women worldwide. Nonetheless, the impact of genetic somatic mutations on glycolysis and oxidative phosphorylation (OXPHOS) genes in BC remains unclear. To fill these gaps, the mutational profiles of 205 screened genes related to glycolysis and OXPHOS in 968 individuals with BC from The Cancer Genome Atlas (TCGA) project were performed. We carried out analyses to characterize the mutational profile of BC, assess the clonality of tumors, identify somatic mutation co-occurrence, and predict the pathogenicity of these alterations. In total, 408 mutations in 132 genes related to the glycolysis and OXPHOS pathways were detected. The *PGK1*, *PC*, *PCK1*, *HK1*, *DONSON*, *GPD1*, *NDUFS1*, and *FOXRED1* genes are also associated with the tumorigenesis process in other types of cancer, as are the genes *BRCA1*, *BRCA2*, and *HMCN1,* which had been previously described as oncogenes in BC, with whom the target genes of this work were associated. Seven mutations were identified and highlighted due to the high pathogenicity, which are present in more than one of our results and are documented in the literature as being correlated with other diseases. These mutations are rs267606829 (*FOXRED1*), COSV53860306 (*HK1*), rs201634181 (*NDUFS1*), rs774052186 (*DONSON*), rs119103242 (*PC*), rs1436643226 (*PC*), and rs104894677 (*ETFB*). They could be further investigated as potential biomarkers for diagnosis, prognosis, and treatment of BC patients.

## 1. Introduction

Breast cancer (BC) is the second most common neoplasm worldwide, affecting approximately 2,308,897 people and leading to 665,684 deaths per year, and has the highest mortality rate among women in at least 150 countries [1,2]. As a heterogeneous disease with a broad spectrum influenced by the environment and intrinsic biological characteristics [3], the investigation of understudied factors that could impact proliferative pathways, such as glycolysis and oxidative phosphorylation (OXPHOS), deserves attention due to their potential relevance to this condition [4,5].

Both glycolysis and OXPHOS are metabolic pathways regulated by the mitochondria, organelles associated with different cancer hallmarks, but not well known in BC [4,5,6,7]. For glycolysis and OXPHOS to occur properly, the nuclear genome must be involved, highlighting the importance of mitonuclear communication [8]. The connection enables various cellular pathways and processes, including metabolism, stress response, and epigenetics. When both compartments function normally, glycolysis and OXPHOS operate correctly [8,9]. This process leads to the formation of approximately 30 enzymes involved in glycolysis and 80 subunits that form OXPHOS complexes, with more than 120 participating genes [9].

Related to the functioning of these pathways, the glycolytic mechanism starts with the transport of glucose by proteins called GLUTs. Then, it is either stored as glycogen (glycogenolysis) or is catabolized to form energy (glycolysis), producing pyruvate for the TCA (tricarboxylic acid cycle) and NADH for OXPHOS [10,11,12]. OXPHOS occurs via five complexes in the inner membrane of mitochondria. Complex I (NADH dehydrogenase/NADH:ubiquinone reductase), complex II (succinate dehydrogenase), complex III (cytochrome c reductase), and complex IV (Cytochrome C Oxidase) [13,14] participate in the generation of mitochondrial energy with their general functions in the OXPHOS, allowing complex V (ATP synthase) to act as a molecular driving force [15].

Alterations in these structures are associated with multifactorial diseases such as cancer. Dysregulation of aerobic metabolism can generate thermogenic imbalance, oxidative stress, defects in calcium metabolism, cellular inflammation, hydroelectrochemical imbalance, and cell death, among others, which can lead to a malignant progression in cancer [16]. One of the phenomena found in most cancers is called the “Warburg effect”, in which the concentration of energy generated is directed toward glycolytic metabolism to the detriment of the OXPHOS process even in aerobic conditions. This results in a basal level of mitochondrial energy production and high lactate levels, supporting the growth, proliferation, survival, and resistance of the tumor [17].

However, some types of neoplasms may not follow this pattern. One example is BC, where the nature of the metabolic imbalance in energy metabolism remains unclear [18,19]. Some pathways linking BC to the Warburg effect are unknown, including the impact of somatic mutations, which vary across cancer types and in BC specifically [20]. While somatic mutations in tumor driver genes like *TP53* and *PIK3CA* are well studied, those in glycolysis and OXPHOS genes are less discussed. Since research on BC often focuses on germline variants, and most cases are not hereditary, further investigation is needed in this area [21,22,23].

Variants in glycolytic and OXPHOS functions have been related to the disturbance or deregulation of the normal functioning of the breast, contributing to the development and progression of BC, being associated with progression, antitherapeutic effects, and also invasion [24]. However, studies of glycolytic and OXPHOS genes are lacking, prompting a search for genes associated with this cancer to improve diagnosis, treatment, and prognosis. The limited research in this area may be due to the complexity of research into metabolic pathways, a historical focus on other oncogenic drivers, and the specialized methodologies required to study these pathways effectively [20,25]. Considering such gaps, the aim of this study was to carry out mutational somatic profiling of glycolysis and OXPHOS genes in individuals with BC.

## 2. Results

### 2.1. Overall Characterization of the Sample Studied Within the Survey

In total, 968 BC samples available in TCGA were used, of which it was possible to verify that 957 (98.86%) individuals were females at birth and 11 (1.14%) were males at birth, with a mean age of 58.24 years for females and 60.64 years for males. These data are shown in Supplementary Table S2. In relation to the normality test for these individuals, a *p* value = 5.779 × 10^−5^ was found, which demonstrates the nonnormality of these samples in relation to the age of these individuals. Furthermore, most of these patients were alive, with 835 (86.26%) alive and 133 dead (13.74%). In terms of tumor information, the majority were samples without metastasis—M0 (83.37%), with a tumor size of T2 (58.67%) and lymph node infiltration of N0 (46.60%). The most common stage was stage II (57.34%) followed by stage III (21.80%), which had a greater ratio of aggressiveness and permissiveness to stage IV, the most severe.

In addition, with regard to ethnicity, the majority were white individuals (670, 69.22%), followed by black or African Americans (157, 16.21%). In terms of histological characteristics, the lobular carcinoma (unspecified—151; 15.60%) and infiltrating ductal carcinoma (unspecified—717; 74.06%) subtypes were the most common within the cohort studied. Both can be named Invasive Carcinoma Without Special Type (NST). As for the age group, most of them were between 26 and 49 years old, with 264 samples (27.27%), which is considered an early onset of BC. The most common molecular subtype was Luminal A (48.14%), followed by TNBC (19.94%) and Luminal B (19.42%).

Most of them are in line with the literature and epidemiological cases of BC, where women are mainly affected by this disease, and it is therefore considered a public health problem for this group [26]. Most of the individuals were in an age group below the average of 62 years, which is considered the diagnostic standard for women with BC, our average was approximately 58 for women, which reveals the possibility of being affected by higher groups [27]. In addition to the clinical characteristics of the tumor (TNM classification), Stage II is the most common, which is supported by the literature [28]. The histological pattern is also in line with what is found in most individuals with BC since infiltrating ductal carcinoma is the most common form. Similarly, for the molecular subtypes, luminal A is the most common among the BC population, as observed in our cohort [27,28,29,30].

### 2.2. General Characterization of the Variants Found in the OXPHOS and Glycolysis Genes

**Figure 1:**
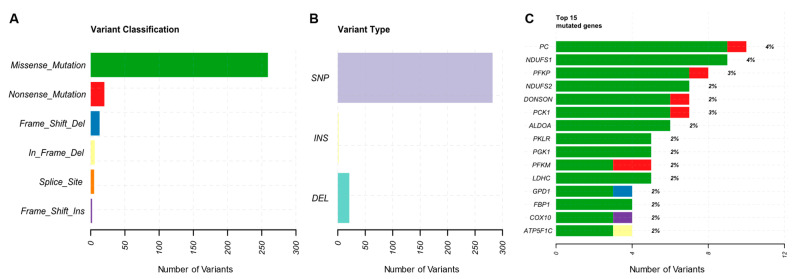
Overview of the glycolysis and oxidative phosphorylation gene variant profile. (**A**) List of the number of variants per type of variant found in the genes targeted in this study. (**B**) Classification of the variants found in general groupings. (**C**) Genes with the highest number of alterations.

Overall, 408 variants (or mutations) were found in genes of glycolysis and OXPHOS in 249 out of 968 samples. After removing duplicated mutations (present in more than one sample), the 398 remaining were assigned to 132 genes, of which 33 of these genes were related to glycolysis and 99 to OXPHOS. A higher alteration rate was observed in glycolysis genes, with approximately 4.4 variants per gene in glycolysis (n [number of variants] = 145 variants), while in OXPHOS, the rate was lower, with approximately 2.7 variants per gene (n = 263 variants). According to the characterization of the variants, 251 (63.07%) mutations were missense, 102 (25.63%) silent (including mutations in splicing regions, mutations in intronic regions, 5′FLANK and 5′UTR regions), 21 (5.27%) frameshift INDELs (19 deletions and 2 insertions), 19 (4.77%) nonsense mutations, and 5 (1.26%) splice site mutations. Missense mutations were the most common within the sample used in this study, and their greater presence in relation to the silent type is worth noting, as shown in Figure 1A with information on the types of change. This research identified single-nucleotide variants (SNVs/SNPs), insertions, and deletions (INS/DEL), present in Figure 1B. Figure 1 summarizes the information on the variants found in this study.

Of the SNVs found, 225 were transitions (C > T: n [number of variants with this modification] = 98, T > C: n = 21, A > G: n = 23, G > A: n = 83) and 150 were transversions (C > A: n = 21, C > G: n = 37, T > A: n = 6, T > G: n = 12, A > C: n = 9, A > T: n = 4, G > C: n = 33, G > T: n = 28).

**Figure 2:**
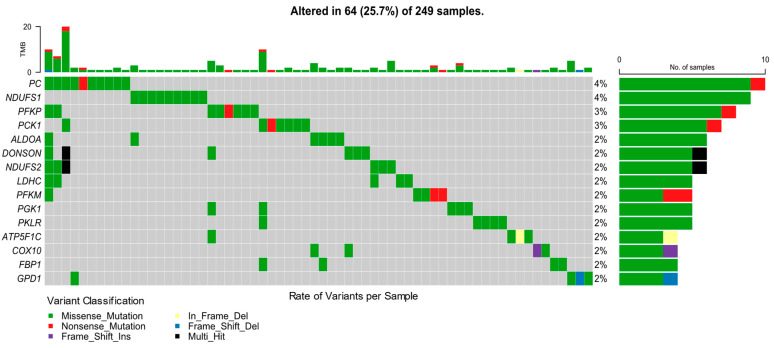
Mutational profile of glycolysis and oxidative phosphorylation genes. Samples affected by mutations in selected genes and the mutation rate per sample or tumor mutation burden (TMB). The nomenclature of the type of mutation and its respective color are indicated below.

As seen in Figure 1C, along with the information on the variants, the gene with the highest number of alterations was *PC* (n [number of variants in this gene] = 13), followed by *PFKP* (n = 10) and *NDUFS1* (n = 9). Notably, there was only one variant present in *NDUFS1* in a total of nine samples, which was the most frequent mutation in this study. Other affected genes included *PFKM* (n = 9), *DONSON* (n = 8), *HK1* (n = 7), *PCK1* (n = 7), *NDUFS2* (n = 7), *ALDOA* (n = 6), and *PGK1* (n = 6). Figure 1C shows the mutation rate per gene, where silent mutations have already been removed, mainly to show potentially pathogenic genes in our cohort and where other potential genes could be identified (*PKLR*, *LDHC*, *GPD1*, *FBP1*, and *COX10*). Figure 2 shows the characterization of variants per sample, highlighting the estimate of tumor mutation burden (TMB), in which sample “TCGA-AN-A046” presents the highest rate, presenting 26 variants in glycolysis and OXPHOS genes.

Based on this, the analysis of the clonality and subclonality of the genes with the highest number of possibly pathogenic variants was carried out using the VAF (variant allele frequency, that is, the frequency of the mutated allele in the sequenced tissue). VAF is a methodology that uses the ratio of sequence reads supporting the presence of the variant allele in the studied tissue compared to the reference allele in the reference genome used, which was GRCh38.p14-hg38. The number of variant alleles is divided by the sum of the variant allele and the reference allele. A high VAF may indicate the presence of the mutation in the initial formation of the tumor. In this work, it was determined that variants greater than 0.5 have a higher clonal potential, while those less than 0.5 are subclonal variants, based on the material provided by the MAFTools package (version 2.22.0) [31], which indicates 0.5 as a regular cutoff to studies involved in MAF file analyses, as well as the methodologies of Li et al. [32] and Boscolo et al., [33], which also indicate a cutoff of 0.5 to distinguish between clonal and subclonal mutations.

**Figure 3:**
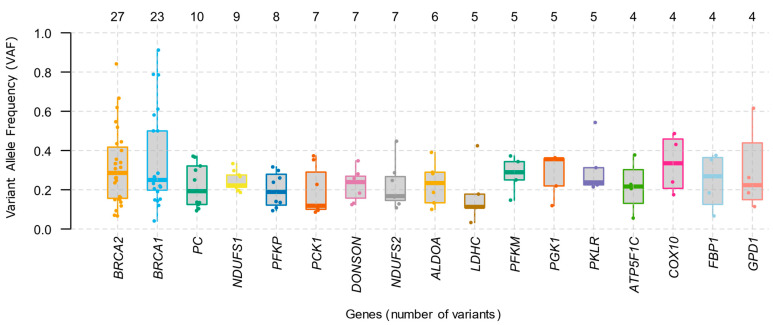
Frequency ratio of the mutated allele. This graph represents the presence of the mutated allele within sequenced somatic tissue based on the number of copies found in the tumor sequencing compared to the reference sequencing. It also represents the sequenced tissue and clonality and subclonality ratio of the variants found in the genes with the highest number of alterations.

In Figure 3, a variant (rs1363038579, 548C>T) in the *GPD1* gene with a VAF greater than 0.6 was identified, indicating that this variant has a possible clonal relationship, as it is present in most of the mutated tissue and is, therefore, possibly related to the onset of the tumor. Other variants are in the *PKLR* gene (c.483C>G) and *PFKM* (c.2118T>C), with a VAF of 0.5, which can also be highlighted as a possible clonal variant. The other genes showed variants with VAF below 0.5, indicating that they are probably subclonal; in other words, they probably occurred when the tumor was already formed. The single variant in the *NDUFS1* (rs201634181) gene found in nine individuals also has subclonal aspects. *BRCA1* and *BRCA2* are included in Figure 3 as a control VAF profile of known breast cancer-associated genes. It was observed that genes such as *GPD1*, *FBP1*, *COX10*, *PFKM,* and *PGK1* obtained a median similar to *BRCA1* and *BRCA2*.

### 2.3. Genetic Interaction between the Genes with the Highest Number of Variants

**Figure 4:**
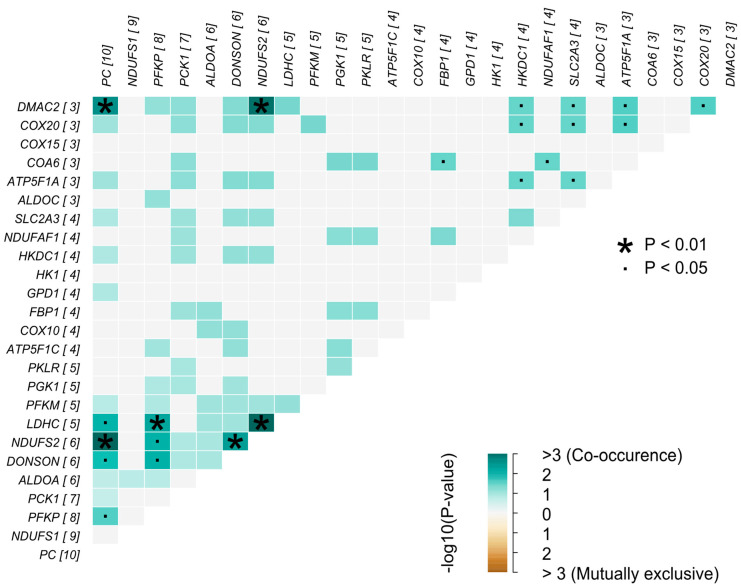
Somatic interaction of glycolysis and oxidative phosphorylation genes with the highest variants. The graph shows the genes and the number of variants found in them in brackets, as well as somatic mutation interaction of the highest number of variants of glycolysis and oxidative phosphorylation genes, looking for genes that have co-occurring variants or mutually exclusive variants. This step consisted mainly of indicating the target genes that are related between or within the two different pathways.

Landscapes of somatic mutation were characterized in co-occurrence for OXPHOS and glycolysis from Fisher’s test for the distribution of conditions. The analysis of somatic interactions based on genes was conducted through two perspectives: examining mutually exclusive events and identifying co-occurring events. In the first analysis, rarer events often manifest as identical mutations appearing in different samples, suggesting that these variants may be impacting a specific pathway or even the tumor process itself, which is why they are mutually exclusive. Conversely, in the context of co-occurring events, it is possible to find mutated genes that appear together in certain samples without the presence of rare events. This indicates that these genes may be involved in similar tumor processes and function synergistically [34]. This analysis is illustrated in Figure 4.

As shown in Figure 4, significant (padj < 0.05) co-occurrence and possible risk genes were identified with odds ratios (ORs). Interactions between the *LDHC* and *NDUFS2* genes (padj = 0.001787961, OR = 414.5155499), *NDUFS2* and *PC* (padj = 0.019069504, OR = 129.1663236), and between *DMAC2* and *NDUFS2* (padj = 0.029066690, OR = 416.4834156) indeed present significant co-occurrence events. Interactions between the OXPHOS and glycolysis genes were greater than 1, which reveals a possible risk of BC in individuals with co-occurrence of alterations in these genes. The OR performed by MAFTools was based on the chances of observing the variants in the BC group compared to the wild-type group. In this research, the wild-type group is considered to be samples with no mutations in either of the analyzed genes. No events of exclusive occurrence between such genes were observed.

**Figure 5:**
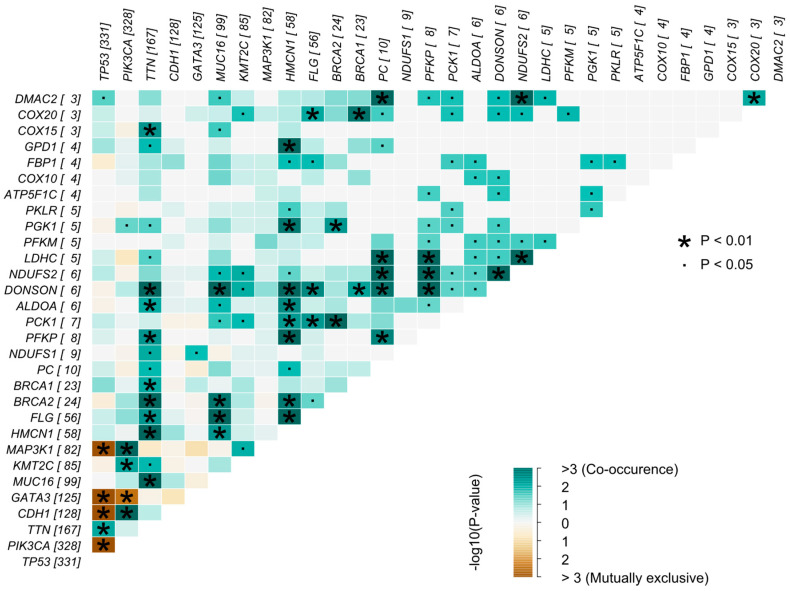
Somatic mutation interactions compared to genes with the highest number of variants. Somatic interactions between the genes with the highest number of variants in the TCGA database and genes associated with glycolysis and oxidative phosphorylation in search of genes with co-occurring variants or mutually exclusive variants. It is possible that these new relationships are maintained in relation to our selected target genes seen in the previous analysis, but here some of them interact with some genes such as *BRCA1*, *BRCA2*, *MUC16*, *HMCN1*, and *TTN*.

The interaction of alterations among the genes with the highest number of variants in BC samples from TCGA revealed significant co-occurrence after adjustment between *DONSON* and *MUC16* (padj = 1.054509 × 10^−3^); *DONSON* and *HMCN1* (padj = 2.661866 × 10^−3^); *PCK1* and *BRCA2* (padj = 6.001709 × 10^−3^); *PFKP* and *HMCN1* (padj = 8.502469 × 10^−3^); *DONSON* and *TTN* (padj = 9.120586 × 10^−3^); *GPD1* and *HMCN1* (padj = 9.283847 × 10^−3^); *BRCA1* and *COX20* (padj = 1.672530 × 10^−2^); *PGK1* and *HMCN1* (padj = 1.876325 × 10^−2^); and *BRCA2* and *PGK1* (padj = 4.526388 × 10^−2^). All interactions between the OXPHOS and glycolysis genes and the genes with the highest number of variants in the TCGA database had ORs greater than 1, which reveals a possible risk of BC in individuals with co-occurrence of alterations in these genes. *BRCA1* and *BRCA2* were highlighted because of their association with HBC. No event of mutual exclusivity of these genes was observed apart from the genes having many variants in TCGA such as *TP53*, as shown in Figure 5. Supplementary Table S3 shows all the information about the interactions between these genes.

### 2.4. Clinical Enrichment of Samples by the Groupwise Comparison

Clinical enrichment was based on the number of variants found per gene in clinical groups. At this stage of the analysis, there was no significant relationship with any of the clinical characteristics presented. Table 1 details the main groupwise comparisons in our cohort, so we just show the top two groupwise. Regarding sex, no gene was found to be significantly enriched. It is worth noting that variants were found in both males and females, although BC is a cancer predominantly found in females. Vital status also showed no significant relationship with any group, as well as metastasis characteristics, tumor size, and estrogen receptor status. In terms of ethnicity, a *p* value close to significance was observed (*p* value = 0.05641932) in relation to *PFKP* for white groups. However, after adjusting (or employing a correction—term to be used in this study for *p*-value adjustment) the *p* value for multiple comparisons (padj or BH = 1), this result was not significant, and the same was observed for this gene in the lymph node infiltration enrichment data.

This situation was also present in the enrichment for tumor stage, where the *DONSON* gene showed a significant *p* value (*p* value = 0.04092777), but after correction, it was no longer associated with BC stage I (padj = 1). In the comparison by histological type, the *NDUFS1* gene was significantly associated (*p* value = 0.01620346) at first with the infiltrating duct mixed with other types of carcinoma, but not after correction (padj = 0.8433735), while the *ALDOA* gene was significantly associated (*p* value = 0.02409639) with the intraductal papillary adenocarcinoma with the invasion type, but also not after correction (padj = 0.8433735). Although it did not appear among the clinical analyses, it is important to mention that the *NDUFS1* variant is present only in women and affects a 36-year-old person.

The age group was subdivided based on the first, second, third, and fourth quartile groups, and a significant association was found (*p*-value = 0.02144538) in the enrichment of the *PCK1* gene with the 68 to 90 age group, although it was no longer present after correction (padj = 0.6004707). Finally, for the molecular subtypes, the *NDUFS2* gene was significantly associated (*p* value = 0.04406721) with the TNBC subtype, but not after correction (padj = 0.9799946). One of the limitations of this research is the small sample size and the large number of North American and European participants. This highlights the need for this type of research to be carried out with larger and more ethnically diverse groups. In addition, the presence of unbalanced groups (such as the number of men with variants) in this study supports the essential research on more homogeneous groups that include a sufficient number of males, since part of this analysis may present biased conditions. However, it is worth mentioning that as BC is more prevalent in women, it is also worth noting that mutations in these genes may be characteristic of this group and therefore deserve to be confirmed.

### 2.5. Prediction of the Pathogenicity of the Variants

The annotation by pathogenicity prediction conducted by eight predictors is shown in Table S4 of Supplementary Material S1. Ada and PROVEAN predictors are available in this material, but we will highlight their main results here when appropriate. Regarding splicing variants and *NDUFS1* (most present variant in our cohort), four variants deserve attention: rs771003498 (*ATP5MC2*.118-1G>A, ada: pathogenic (0.99998)); COSV65781836 (*COX4I2*.247+1G>A, ada: pathogenic (0.99996)); COSV99749106 (*UQCRB*.19+2T>C, ada: pathogenic (0.96604)); rs201634181 (*NDUFS1*.1868T>G, SIFT: deleterious (0), PolyPhen: possibly damaging (0.897), ada: pathogenic (0.838560013), cadd: 29.6, Bayes: damaging, gerp: 5.63, LOEUF: 0.800, PROVEAN: damaging). The COSV65781836 and COSV99749106 variants (not yet registered in the dbSNP) shown here are noteworthy for having ada close to 1 and CADD above 30, indicating that the pathogenicity of these variants has a high probability of affecting splicing. The LOEUF of these variants was also high, indicating that they are not highly conserved and are likely to be altered. The main variants are highlighted in Table 2.

Overall, for the other variants, we decided to proceed by finding common predictions in the BayesDEL and CADD databases. Thus, the variants with the greatest possibility of being affected were *HK1*.2365C>T (COSV53860306); *PFKM*.2057G>A (COSV56656860); *PFKM.*2285C>A (COSV56657886); *DONSON*.994G>T (COSV51655479); and *PC*.3026C>A (COSV58992778). Among these five alterations, the alteration in the *HK* gene has an increased pathogenicity rate according to the database where it is present and has high conservation (LOUEF < 0.35 and LOEUF between 4 and 6); moreover, this gene was not among the genes with the highest number of variants. None of these variants are already registered in dbSNP.

Other possible pathogenic variants were filtered according to eve, as they were not as conserved (LOUEF > 0.35 < 1), but at the threshold of being considered conserved: *NDUFS2*.719T>G (COSV63487523, PROVEAN: damaging); *COX10*.900G>C (COSV55404425, PROVEAN: damaging); and *DONSON*.631C>T (rs774052186, PROVEAN: damaging). None of these variants have such a low conservation, but they can still be considered possible variants to be further studied, mainly because they show similarities of prediction in all considered databases. The rs774052186 variant has already been reported in the literature.

Regarding other variants documented in the global literature, they exhibit general characteristics of pathogenicity: *PC*.1748G>A (rs119103242, PROVEAN: damaging); *PC*.584C>T (rs1436643226, PROVEAN: damaging); *ETFB*.491G>A (rs104894677, PROVEAN: damaging); *FOXRED1*.694C>T (rs267606829, PROVEAN: damaging). All of them are reported in the literature and have already been correlated with some kind of disease, not necessarily cancer, highlighting the importance of these findings.

## 3. Discussion

In our main results, we highlight genes such as *PGK1*, *PFKM*, *PKLR*, *PC*, *PFKP*, *ALDOA*, *HK1*, and *PCK1* in relation to glycolysis; as for the OXPHOS genes, we emphasize *DONSON*, *NDUFS1*, *NDUFS2*, and *GPD1*. Overall, genes such as *PC*, *PFKP*, *PFKM*, *PCK1*, *DONSON,* and *NDUFS1* are already associated in the literature with proliferation, invasion, migration, metabolic reprogramming, metastasis, and poor prognosis in BC [35,36,37,38,39,40,41,42,43].

The gene with the highest number of variants or mutations, *PC*, is related to oxaloacetate formation [44], and its variants were associated with metastasis from upregulated glycolysis [45]. Two variants (*PC:*c.584C>T and *PC*:c.1748G>A) found in the *PC* have been previously reported to have defects in OXPHOS, high pathogenicity in children, and the allosteric regulation of *PC* [46,47]. Both variants are characterized by fluctuating levels of conservation (which are neither firmly established nor significantly lacking). This corroborates our work by showing that the mutations found in this conserved region may be associated with BC and deserve validation. However, none of these mutations were clonal, which could limit their disruptive role after cancer formation for our variants and may not have a direct impact in BC.

In BC, mutations in the *ALDOA* gene have been found to be more frequent than in other types of cancer, although they may not be associated with the clinical condition [48,49]. Thus, variants identified had a small initial relationship with the histological subtype “intraductal papillary adenocarcinoma with invasion” and ethnicity “not reported”, which corroborates that it is not in fact related to clinical conditions in BC, but merits further investigation.

Mutations in *PFKP* and *PFKM* are related to basal glycolysis, glycogen storage diseases, and metastatic progression, and its loss decreased tumor proliferation and progression in cancer, which suggests pathogenic variants in these genes could be associated with BC, especially since *PFKM* had a variant with a VAF above 0.5, potentially indicating its key role in initial tumor formation, which deserves attention [36,37,38,50]. We suggest that this mutation may serve as a key prognostic element in BC, given its clonal nature and potential to improve prognostic accuracy.

The genes *PFKP*, *PFKM,* and *PGK1* were associated with oncogenes that could reinforce the Warburg effect and tumor progression [51,52,53,54]. In our report, this relationship indicates that the related hub genes have the tumorigenesis function and suggests that our target genes may not be the primary driver, but rather a supportive factor for the hub gene, supporting the idea that these could act as factors in BC [55]. The loss of *PGK1* is associated with the inhibition of carcinogenesis and is also a marker of progression in gastric, oral, and liver cancer [56]. This type of information suggests that this gene can be further investigated as a treatment and prognosis driver in BC.

Mutations in the *PKLR* gene are associated with overall cellular dysfunction due to disturbances in glycolytic metabolism [57,58,59,60] and have been linked to gastric cancer, although their specific influence remains unclear. This suggests that the mutations in *PKLR* identified in this study warrant further investigation. Like *PFKM* and *GPD1*, *PKLR* also has a variant with a VAF above 0.5, indicating a potential relationship with the initial stages of tumor formation and possible glycolytic dysfunction. These variants are some of the key findings of our study and should be explored further, as they may be crucial elements in BC prognosis.

In our study, *HK1* had a high conservation ratio and pathogenic variant, and its loss leads to tumor cells by deregulation and activation of apoptosis, corroborating our study since the mutations found here are pathogenic. This would then cause the loss of *HK1* and then lead to tumor death, corroborating the treatment and prognosis of BC. However, it is important to mention that this suggestion is based on its function and involvement in other types of cancer like liver tumors, where it is related to aerobic glycolysis, which interacts with mitochondrial calcium metabolism and apoptosis [60,61]. In addition, *PCK1* is the main controller of gluconeogenesis [62]. Variants in this gene report dysfunction in liver tissue, as well as short stature and neurocognitive disorders [63,64], which suggests that its loss of function from pathogenic variants may be associated with decreased glycolysis and the Warburg effect, and reduced tumor growth.

The *DONSON* gene in BC is linked to mitochondrial apoptotic pathways, and this gene is directly involved in the process of repairing and stabilizing DNA replication, but its action on metabolic pathways is indirect [39,40]. *DONSON* may be associated with the action of the DNA Damage Response (DDR), in which there is an increase in OXPHOS as a compensatory mechanism involving a reduction in genotoxic stress [65,66,67]. The variant reported in this gene (*DONSON*:c.631C>T) was related to severe cases of Meier–Gorlin syndrome (MGS), where one of its clinical characteristics is abnormal breast formation [68,69,70]. This enables malformation and tangling between the *DONSON* proteins, causing possible problems for the tissue, which corroborates our results, indicating that this variant may be associated in a pathogenic way with breast function [70]. In our study, this gene demonstrates its link with mutations in oncogenes associated with HBC [71,72,73,74,75], suggesting that this gene may be associated with the cell cycle and mitochondrial function, corroborating that its function may be beyond the impact of somatic mutations [76].

*NDUFS1*, the gene with the most present variant (*NDUFS1*.1868T>G) in different samples in our cohort and not yet reported, and *NDUFS2* have already been linked to cancer, mitochondrial diseases and being associated with oncogenes [77,78,79,80,81,82,83]. Variants in this gene in colorectal tumor cells lead to a loss of OXPHOS and result in disordered proliferation, likely due to *NDUFS1′s* essential role in initiating phosphorylation [41]. *NDUFS1* is the largest subunit of Complex I and performs the primary dehydrogenase function of this complex, facilitating NADH oxidation and initiating the OXPHOS. Thus, *NDUFS1* also interacts with genes involved in metabolic processes, such as *SOAT1* and *HLA-B* [78,79,80,81,82,83]. The variant found in our study exhibits high pathogenicity and a subclonal relationship. However, there is limited information regarding this variant, suggesting it may have a random effect or just cause dysfunction that remains undefined, necessitating further studies and validated essays in the future [42,43].

*GPD1* is considered an anti-tumor gene, and its overexpression inhibits proliferation, migration, and invasion [84,85,86]. One of the variants highlighted in our research (*GPD1*:c.548C>T) has a high clonality ratio and may be associated with the loss of function of GPD1 at the beginning of tumor formation, thus leading to cancer aggressiveness [86]. This particular variant deserves attention since it has aspects related to tumor formation and could be suggested as a possible prognostic factor in BC.

In BC, the *ETFB* variants, related to the normal functioning of OXPHOS, have already been associated with a cardiotoxic effect [87], and their disfunction was related to lung cancer [88] and acute myeloid leukemia [89,90]. Our variant found in this article has already been reported (*ETFB*:491G>A) in patients with Glutaric acidemia type II, with poor prognosis in all individuals with the alteration [91], suggesting it may lead to destabilization of the protein in BC.

The mutation (*FOXRED1*:694C>T) is associated with Complex I deficiency, which leads to mitochondrial dysfunction and encephalopathy [92], as well as association with Leigh syndrome and the Warburg effect, due to deficient OXPHOS, the suggestion is that the organism would tend to elevate glycolysis [93,94]. Therefore, dysfunction in a highly conserved gene corroborating our study and highlighting the indication of SNV rs267606829 needs to be studied [94,95,96]. The mutation in the *FOXRED1* gene has been well documented, and as it has already been associated with the Warburg effect, this variant is gaining attention because it has been related to a type of prognostic outcome that may be present in BC.

Among the main genes involved, *PGK1*, *PFKM*, *PKLR*, *ALDOA*, and *HK1* play essential roles in the glycolytic pathway by interacting with each other. *HK1* catalyzes the conversion of glucose transported into cells by the GLUTs into glucose-6-phosphate, which is then transformed into fructose-6-phosphate by *GPI*. *PFKM* subsequently converts it to fructose-1,6-bisphosphate, and *ALDOA* converts this into glyceraldehyde 3-phosphate and dihydroxyacetone phosphate [97,98,99]. *PKLR* functions at the end of glycolysis, converting phosphoenolpyruvate to pyruvate. The *NDUFS1* and *NDUFS2* genes interact by receiving the glycolysis products like NADH [14,80], while *PC* and *PCK1* interact within the gluconeogenesis pathway [97,99]. These gene interactions may suggest that mutations in *NDUFS1* and *NDUFS2* impair NADH oxidation, potentially affecting electron transport efficiency.

From our findings, most of the genes highlighted in the cohort have already been linked in the literature to the development of cancer. We highlight some of these genes (*PC*, *PFKM*, *PGK1*, *PCK1*, *DONSON*, *NDUFS1*, and *GPD1*) as potential candidates for investigation in larger cohorts. It is worth noting that our conclusions are based on bioinformatics analysis, so experimental validation in future studies is necessary. In addition to characterizing the genes with the highest number of variants, we need to identify the most prevalent variant in our cohort and the interaction between variants of glycolysis and OXPHOS genes with oncogenic function genes. The VAF analyses highlight the clonality of these variants, suggesting that these alterations can confer selective advantages to cells, leading to high cell proliferation. We suggest that future studies should include longitudinal analyses of tumor tissue with these variants (in *GPD1*, *PLKR,* and *PFKM*) to understand their clonal evolution and the possible action as tumor driver progression [100,101].

In fact, one of the limitations of this research and suggestions for future studies is the need for more heterogeneous population groups, since we used a database with a majority of white groups, or even research on under-represented groups of individuals such as indigenous peoples, quilombolas, and riverine communities. Considering the lack of population diversity in our study, we encourage future research to focus on under-represented groups, and also collaborations between institutions from different countries. This would help identify common variants among populations and rare variants specific to different populations with BC worldwide. Of the main genes found, only the *PCK1* gene has been reported in under-represented groups. The first was by Abrahams-October et al. [102], who found SNPs in this gene associated with anti-chemotherapy response in the indigenous Nguni population of South Africa, but no association was found. The second is by Rees et al. [103], who indicated that in South Asian populations living in the United Kingdom (UK) with Punjabi ancestry, there was a higher risk of developing type 2 diabetes mellitus (T2DM) from the *PCK1* gene. These studies reinforce this gene in human diseases and should be evaluated and confirmed in BC.

In addition, the prevalence of mutations in the target genes, which vary from 1 to 2% in our cohort, is noteworthy. It is suggested that these mutations may not be the primary cause of tumor development in BC but could be coincidental and might not have a direct role in tumorigenesis. Another point is that these mutations may have a key role underlying other major genes, which may in fact be causing the tumorigenesis [104,105].

The fact must also be that instead of being influenced by somatic mutations, the role of glycolysis and OXPHOS may be more impacted by gene regulation, particularly epigenetics. This perspective allows us to consider additional regulatory mechanisms, such as protein regulation within these metabolic pathways and the functions of non-coding RNAs, including microRNAs. It is also possible that these genes, despite having low mutation rates in tumor tissue, are conserved due to selective pressure because of their essential roles, such as energy production functions [106]. This suggests that these functions deserve to be better clarified in future studies.

In addition, *PCK1*, *DONSON*, *PFKP*, *PGK1,* and *GPD1* genes were also associated with oncogenes in our study, such as *BRCA1*, *BRCA2*, *HMCN1,* and *TTN*, which have already been reported to be mainly associated with HBC. We recommend that future studies also check these genes for their function in glycolysis and OXPHOS in individuals with this type of BC, especially *DONSON*, which is already related to traits of apoptotic mitochondrial function and cell cycle. These notes further support our suggestion that these somatic mutations may not be key elements in such genes in BC, but that they may act as internal elements in it.

## 4. Materials and Methods

### 4.1. Data Availability and Ethical Aspects

Nine hundred and sixty-eight primary tumor tissue samples from individuals with BC were investigated and are available in The Cancer Genome Atlas database (TCGA—https://portal.gdc.cancer.gov/projects/TCGA-BRCA, accessed on 24 March 2024, version 41.0 and dbGaP Study Accession identification—phs000178). The MAF (Mutation Annotation Format) files from open access BC samples were analyzed for the analysis, and no closed-access data were used. The data were previously processed by TCGA according to its workflow (available at https://docs.gdc.cancer.gov/Data/File_Formats/MAF_Format/, accessed on 24 March 2024, version 1.0). There was no need to submit this research to the Research Ethics Committee, and this study respects the Nuremberg Code and the Declaration of Helsinki.

### 4.2. Data Processing

The data were downloaded from TCGA, analyzed via RStudio IDE (R version 4.4.0 and RStudio version 2024.09.1+394) [107], and then processed using the “tidyverse”(version 2.0.0) [108], “R.utils” (version 2.12.3) [109], and “data.table” (version 1.15.4) [110] packages. These steps were carried out to ensure that only somatic mutations were analyzed, that duplicated individuals and mutations per sample were removed, and that clinical information could be appended per sample.

### 4.3. Standardization and Analysis of Clinical Data

As a first step in analyzing the clinical data, the clinical characteristics provided by TCGA were standardized in this study to reduce statistical bias and simplify the multitude of classifications. Thus, the data on lymph node infiltration, tumor stage, and tumor size were standardized into their main categories following the American Joint Committee on Cancer (AJCC) standardization [111]. Age was grouped into age groups based on the quartiles of the overall age grouping. Both the normal-like and basal-like molecular subtypes were merged as triple-negative breast cancer (TNBC) subtypes. Age, sex, ethnicity, metastasis, lymph node infiltration, tumor stage, tumor size, histological subtype, and molecular subtype were then used to analyze the clinical data.

### 4.4. Analysis of Oxidative Phosphorylation and Glycolysis Genes

The MitoXplorer 2.0 platform [112] was used to select the genes associated with energy metabolism. MitoXplorer is a platform for the systematic analysis of genes involved in mitochondrial functions. For the selection of specific genes, we filtered the genes related to the terms “Glycolysis” and “Oxidative Phosphorylation”. These terms included regulatory genes and structural genes. Only nuclear genes directly related to the glycolysis and oxidative phosphorylation process were included here, and no genes from the mitochondrial genome were included. Considering these criteria, a total of 205 genes were selected (Table S1 of Supplementary Materials).

### 4.5. Variant Analysis

The R environment through R Studio IDE [107] was used to search for variants within the MAF files, using internal functions to screen and index variants in the selected genes. Single-nucleotide variants (SNVs) and insertions and deletions (IN/DEL) were selected for downstream analysis. The “maftools” package (version 2.22.0) [31] was used for general summarization of mutations (e.g., counting mutations, counting per mutation types, and characterization of transition or transversion mutation), mutational landscape across samples (this analysis identifies samples with a greater number of alterations in the glycolysis and OXPHOS genes, samples with more than one alteration per gene, or samples with multiple altered genes among our target genes), frequency of the variant allele in tumor tissue (with a cutoff higher than 0.5 for clonality variants), clinical interaction between genes and their conditions (groupwise comparison), and somatic interaction between target genes with a large number of alterations.

Somatic interaction seeks to identify variants in genes that occur in common, either as co-occurrences (without exclusive variants—potential target disease genes that work together in a biological pathway) or mutually exclusive (there are exclusive variants that appear in common in different individuals—potential variants between two potential targets genes). This analysis was divided into two parts in this research: (1) The first was carried out in order to identify co-occurring or mutually exclusive characteristics in the 25 genes with the greatest number of variants within the glycolysis and OXPHOS genes. (2) For the second, two criteria were used: being among the top 10 genes with the greatest number of variants within the TCGA BC database; and if they are already reported in relation to the development and formation of the BC, the *BRCA1* and *BRCA2* genes are already reported to be associated with the formation of hereditary breast cancer (HBC) and may also be associated with somatic tumor formation [113,114], being selected to verify interactions with our target genes. Thus, 12 genes (*TP53, PIK3CA, CDH1, TTN, GATA3, MUC16, KMT2C, MAP3K1, HMCN1, FLG, BRCA1,* and *BRCA2*) were selected to determine whether they have somatic interactions with the 15 genes with the greatest number of variants among the glycolysis and OXPHOS genes.

### 4.6. Pathogenicity Prediction

The screened variants were annotated to GnomAD (Genome Aggregation Database) [115], dbSNP (Database of Single-Nucleotide Polymorphisms) from the National Center for Biotechnology Information (NCBI) [116], and EnsemblVEP [117]. All three databases contain information on genetic variants and were used to determine whether these alterations were already described in the literature. The reference genome used in this research was GRCh38.

With the information collected, the variants were identified with their respective HGVS (Human Genome Variation Society) nomenclature to EnsemblVEP [117], for pathogenicity prediction from PolyPhen 2.0 [118], SIFT [119], Eve [120], Ada [121], CADD [122], BayesDel [123], Gerp [124], LOEUF [125], and Provean [126]. Ada is a pathogenicity predictor used for splicing variants, and it was first used to characterize under-represented splicing site variants (Ada > 0.9) that were predicted in the CADD and LOEUF databases at any level of prediction. The second filter was based on CADD (score > 35) and BayesDel (prediction = damaging). Both are databases that consider the pathogenic relationship in the position where the alteration was present, as well as conservation aspects (location of the alteration and how the product could be damaged). This was also done because the other prediction databases do not have information on nonsense alterations.

The third filter was based on CADD (score 30 to 35) and Bayes (prediction = damaging) and is now mainly associated with missense variants, thus including SIFT, PolyPhen, Eve, GERP, LOEUF, and PROVEAN. SIFT and PolyPhen consider the pathogenicity relationship only for nonsynonymous alterations, but have little information for nonsense and frameshift alterations, so they have been complemented, for which the lowest and highest levels have been considered, respectively (SIFT: deleterious < 0.01, PolyPhen: damaging > 0.7), Eve (damaging > 0.9), GERP (between 4 and 6 = damaging). For all the filters made, the LOEUF was standardized to < 0.350 because of the sites of high conservation and consequently high damage. PROVEAN was also used, taking into account its prediction as damaging, which also takes conservation into account to verify damage. Variants with similar predictions were selected. The final filter was used for variants that had already been dated in the PubMed literature. In this research, the terms “variants” and “mutations” were treated equally.

### 4.7. Statistical Analysis

Fisher’s exact test was applied to check the distribution of conditions. The *p*-values were adjusted using the Benjamini–Hochberg (BH) correction method with alpha at 0.05 to indicate statistical significance, and the results are presented here as adjusted *p*-value (padj).

## 5. Conclusions

Most of the genes highlighted here are associated with the control of glycolysis and OXPHOS, and some of these alterations in a glycolysis gene (mutation or loss of the gene) can reduce the proliferation and aggressiveness of the tumor. In the case of OXPHOS, the loss of these genes helps to increase aggressiveness. Thus, we indicate seven variants—rs267606829 (*FOXRED1*), COSV53860306 (*HK1*), rs774052186 (*DONSON*), rs201634181 (*NDUFS1*), rs119103242 (*PC*), rs1436643226 (*PC*), and rs104894677 (*ETFB*)—to be studied and confirmed in large populations, with emphasis on the rs267606829 variant that has already been addressed in several studies focusing on *FOXRED1* function in human diseases. Additionally, the rs201634181 variant was present in many individuals within our cohort and only in women, which deserves attention. Overall, the investigation carried out on the genes with the highest number of variants in OXPHOS and glycolysis, along with literature information, shows that these pathways and genes may not play an intrinsic role in the carcinogenic process in BC in relation to somatic mutations.

## Figures and Tables

**Table 1:** Clinical enrichment between clinical characteristics and target genes.

Gene	Group 1	Group 2	Group Comparison	*p-*Value	OR	BH
***Sex assigned at birth***						
*PC*	Female	Others	9 of 245/1 of 4	0.1520854	0.1170333	1
*PC*	Male	Others	1 of 4/9 of 245	0.1520854	8.5445772	1
***Vital status***						
*PC*	Alive	Others	7 of 216/3 of 33	0.1327041	0.3369989	0.7928395
*PC*	Dead	Others	3 of 33/7 of 216	0.1327041	2.9673687	0.7928395
***Metastasis***						
*PC*	M1	Others	1 of 6/9 of 243	0.2200264	5.1292482	1
*PFKP*	M0	Others	8 of 210/0 of 39	0.3638725	Inf	1
***Tumor size***						
*DONSON*	T1	Others	3 of 61/3 of 188	0.1591165	3.1714430	1
*NDUFS2*	T3	Others	2 of 31/4 of 218	0.1637115	3.6604638	1
***Lymph node infiltration***						
*PFKP*	N1	Others	0 of 80/8 of 169	0.05740682	0.000000	1
*DONSON*	N0	Others	5 of 120/1 of 129	0.10889792	5.5323379	1
***Ethnicity***						
*PFKP*	White	Others	8 of 168/0 of 81	0.05641932	Inf	1
*ALDOA*	Not reported	Others	2 of 29/4 of 220	0.14621725	3.9654698	1
***Breast cancer stage***						
*DONSON*	Stage I	Others	3 of 36/3 of 213	0.04092777	6.2868881	1
*PFKP*	Stage I	Others	3 of 36/5 of 213	0.09303840	3.7523907	1
***Histological type***						
*NDUFS1*	Infiltrating duct mixed with other types of carcinoma	Others	2 of 6/7 of 243	0.01620346	16.2320734	0.8433735
*ALDOA*	Intraductal papillary adenocarcinoma with invasion	Others	1 of 1/5 of 248	0.02409639	Inf	0.8433735
***Age***						
*PCK1*	68–90	Others	5 of 71/2 of 178	0.02144538	6.6062457	0.6004707
*NDUFS2*	68–90	Others	4 of 71/2 of 178	0.05703033	5.2127699	0.7984246
***Molecular subtype***						
*NDUFS2*	TNBC	Others	4 of 66/2 of 183	0.04406721	5.7884424	0.9799946
*ALDOA*	Luminal B	Others	3 of 44/3 of 205	0.06999961	4.8812654	0.9799946
***Estrogen Receptor Status***						
*NDUFS1*	Negative	Others	1 of 96/8 of 153	0.1593507	0.1917227	0.9680924
*NDUFS1*	Positive	Others	7 of 136/2 of 113	0.1886977	2.9996292	0.9680924

OR = odds ratio; Inf = number that cannot be measured or accessed.

**Table 2:** Main variants in pathogenicity prediction, with the main ensemblVEP prediction databases highlighted.

Variant	SIFT	PolyPhen	Eve	CADD	BayesDel	gerp	LOEUF
***Splicing variants***							
*ATP5MC2*:c.118-1G>A	-	-	-	25.5	-	-	1.61
*COX4I2*:c.247+1G>A	-	-	-	34	-	-	1.34
*UQCRB*:c.19+2T>C	-	-	-	31	-	-	0.943
***Highly pathogenic variants (CADD and Bayes)***							
*PFKP*:c.1141C>T	-	-	-	40	Damaging	5.39	0.929
*HK1*:c.2365C>T	-	-	-	48	Damaging	5.29	0.326
*NDUFS3*:c.512G>A	-	-	-	44	Damaging	5.87	1.03
*PC*:c.3026C>A	-	-	-	39	Damaging	4.89	0.435
*PFKM*:c.2057G>A	-	-	-	46	Damaging	05.05	0.866
*PFKM*:c.2285C>A	-	-	-	40	Damaging	05.05	0.866
*OXA1L*:c.556C>T	-	-	-	38	Damaging	5.76	0.808
*ADCK1*:c.403C>T	-	-	-	38	Damaging	5.3	1.27
*GAPDHS*:c.981C>G	-	-	-	35	Damaging	5.5	0.808
*PCK1*:c.942G>A	-	-	-	45	Damaging	5.27	1.29
*DONSON*:c.994G>T	-	-	-	41	Damaging	6.04	0.854
*NDUFV3*:c.302C>G	-	-	-	37	Damaging	6.01	1.87
*UQCR10*:c.112C>T	-	-	-	43	Damaging	5.73	1.71
*UQCR10*:c.112C>T	-	-	-	43	Damaging	5.73	1.71
*COX7B2*:c.229G>T	-	-	-	35	Damaging	4.57	-
*HIGD2A*:c.226C>T	-	-	-	39	Damaging	5.89	1.57
*PGK2*:c.1072G>T	-	-	-	37	Damaging	4.19	1.61
*NDUFA8*:c.283C>T	-	-	-	40	Damaging	5.18	0.724
*SURF1*:c.469C>T	-	-	-	35	Damaging	4.64	1.72
***Variants with a similar factor in most databases and pathogenic***							
*HK1*:c.2680G>C	deleterious low confidence (0)	probably damaging (0.954)	Uncertain (0.55109)	32	Damaging	5.78	0.326
*NDUFS2*:c.719T>G	deleterious low confidence (0)	probably damaging (0.981)	Pathogenic (0.72090)	32	Damaging	5.29	0.560
*NDUFS2*:c.1363G>A	deleterious low confidence (0.01)	probably damaging (0.987)	Uncertain (0.63287)	32	Damaging	5.25	0.560
*GPI*:c.1140G>T	deleterious low confidence (0)	probably damaging (1)	Pathogenic (0.84937)	32	Damaging	5.61	0.561
*COX10*:c.900G>C	deleterious (0)	probably damaging (0.933)	Pathogenic (0.74048)	32	Damaging	4.7	0.611
*NDUFS8*:c.385G>A	deleterious low confidence (0)	possibly damaging (0.797)	Uncertain (0.60533)	32	Damaging	4.53	0.738
***Variants present in the PubMed literature***							
*DONSON:*c.631C>T	deleterious (0)	probably damaging (1)	Pathogenic (0.80402)	33	Damaging	5.99	0.854
*NDUFB9*:c.321G>T	deleterious (0)	probably damaging (0.996)	Pathogenic (0.78166)	32	Damaging	5.43	1.04
*NDUFAF5*:c.375G>C	deleterious (0.02)	benign (0.342)	Uncertain (0.54972)	33	Damaging	5.82	1.20
*ETFB*:c.491G>A	deleterious low confidence (0.02)	possibly damaging (0.832)	Uncertain (0.59339)	28.3	Damaging	5.26	0.825
*PC*:c.1748G>A	deleterious low confidence (0)	probably damaging (1)	Pathogenic (0.92527)	27.2	Damaging	5.53	0.435
*PC*:c.584C>T	deleterious low confidence (0)	probably damaging (1)	Pathogenic (0.91731)	27.7	Damaging	4.99	0.435
*FOXRED1*:c.694C>T	-	-	-	29.7	Damaging	5.77	1.29

-: Not Reported or Not Available.

## Data Availability

The dataset supporting the conclusions of this article is available in The Cancer Genome Atlas repository of identifier dbGaP (study accession number: phs000178) at https://portal.gdc.cancer.gov/projects/TCGA-BRCA, accessed on 24 March 2024, version 41.0.

## Supplementary Materials

The following supporting information can be downloaded at: https://www.mdpi.com/article/10.3390/ijms252312585/s1.
